# The Next Pandemic: Prepare for “Disease X”

**DOI:** 10.5811/westjem.2020.5.48215

**Published:** 2020-06-08

**Authors:** Kenneth V. Iserson

**Affiliations:** The University of Arizona, Department of Emergency Medicine, Tucson, Arizona

The COVID-19 pandemic will, slowly, and with some hiccups and many tragedies, pass into memory. This coronavirus may disappear and later recur, continue endemically under vaccine control, or simply attenuate and vanish.^1^ The economy and healthcare systems will return to a new normal, some parts more quickly than others. Like the multiple plagues humanity has endured since our ancestors gathered into cities, it will generate recriminations for slow and misguided responses, profiteering, and overor underreacting to economic, social, and healthcare events that will, retrospectively, be obvious.^2^ The individuals and organizations most culpable for exacerbating the disaster (e.g., many national and some state political leaders) will escape responsibility while they scapegoat others and try to re-write history. Heroes, whether individuals who helped provide clear risk communication and leadership (e.g., Anthony Fauci, MD, of the National Institutes of Health; Sanjay Gupta, MD, of CNN; and Li Wenliang, MD, who died while trying to notify the world about the pandemic) or groups that persevered in the face of fear and life-threatening danger (e.g., emergency department, intensive care unit, emergency medical services, and other critical healthcare staff and first responders) will emerge. Without fanfare, most will return to their normal jobs, scarred but proud of their efforts. As they have before, pundits and scholars will write endlessly about the pandemic’s cause, effects, and ways to ameliorate the next pandemic’s brutal destruction of lives and ways of life. The problem is, we have done all this before and seem not to have learned the lessons our predecessors taught.

To most people, COVID-19 appears to be an anomaly; it isn’t. The 20^th^ century began with devastating waves of Spanish flu that killed from 50 million to 100 million people worldwide. About one new disease is emerging each year.^1^ Not all have human-to-human transmission, but enough do (e.g., severe acute respiratory syndrome, Middle East respiratory syndrome coronavirus, Ebola virus disease) to scare those tasked with monitoring the world’s health. To highlight the danger and to prioritize research, each year the World Health Organization (WHO) commissions an expert committee to update its list of the most threatening infectious diseases that lack effective treatments or vaccines. The current list (Table 1) contains COVID-19.^3^ That is no surprise, given that the entire world is now focused on that pathogen. What should act as a wake-up call to seriously fund the surveillance of, research into, and treatment of the wide variety of potential pandemic agents is the entity at the end of the short list: Disease X. Since 2015, the WHO has used this designation for a disease that could cause a pandemic due to a pathogen currently unknown to cause human illness. Last year’s Disease X now has a name, COVID-19. The next unknown and unnamed entity may already be lurking.

One might ask: Why don’t we devise a plan to identify such pathogens early and mobilize scientists, the healthcare community, politicians, and the populace to fight these scourges? The answer is, we already have. We know what steps to take to limit a pandemic. WHO, the Centers for Disease Control and Prevention (CDC), and the Departments of Homeland Security and Health and Human Services have produced and disseminated detailed plans.^4^–^7^ After the SARS pandemic, for example, WHO itemized the steps needed to control a pandemic (Table 2). These vital steps were ignored during the initial period of the COVID-19 pandemic.^1^ WHO, chronically underfunded, is saddled with a bloated, slow, and uncoordinated bureaucracy that has to answer to 194 countries. It has been condemned for both overreacting (2009 H1N1 pandemic) and severely underreacting (2014 Ebola epidemic and the COVID-19 pandemic) and for failing to act.^8^–^11^ The CDC is chronically underfunded and has no political power. Academics are voices in the wilderness whose advice is usually sought too late in the process for it to have much effect.

As the COVID-19 threat lessens, politicians will make grand promises to implement plans to stop, or at least to prepare for, the next pandemic. The recovering economy will be too weak at first to support the effort, although more funding will be promised in the future. Politicians will ultimately make changes that are politically expedient and will fail to authorize the changes necessary to produce faster, more flexible responses. The memories of angst and societal disruption during COVID-19 will recede. Our bulwarks against pandemic diseases will remain underfunded and inadequate to the task. Even so, multiple Disease Xs are clearly in our future; we need to be prepared.

## Figures and Tables

**Table 1:** World Health Organization list of emerging diseases for research prioritization.^3^

COVID-19 Crimean-Congo hemorrhagic fever Ebola virus disease and Marburg virus disease Lassa fever Middle East respiratory syndrome coronavirus (MERS-CoV) and severe acute respiratory syndrome (SARS) Nipah and henipaviral diseases Rift Valley fever Zika Disease X

**Table 2:** Lessons learned after SARS: preparation, management, and risk communication.^1^,^6^,^7^,^11^

**More pandemics will appear**. Be prepared. COVID-19 will not be the last new disease to take advantage of modern global conditions. Continued vigilance is vital. Preparation includes enhancing the integration and effectiveness of the public health, healthcare, and emergency management systems through education, supplying adequate provisions, and drills as well as developing incentives (eg, tax credits, identified cost savings) that increase the number of nongovernmental entities engaged in actions that enhance their communities’ health security. **Report cases early**. Global health security requires promptly identifying and reporting cases of any disease with the potential for international spread. Concealing these cases or denying that they exist carries the potential for enormous human suffering and death, loss of international credibility, negative domestic and regional economic impact, and a very real risk the outbreak will spiral out of control. **Alert the world**. As soon as an emerging and transmissible infection is confirmed, international bodies, such as WHO, must issue a global alert through all available communication modalities. **Promote international scientific collaboration**. The world’s scientists, clinicians, and public health experts must act collaboratively to investigate, control, and, if possible, eliminate the disease. **Provide leadership and consistency**. Coordinating messages and policy among federal, state, and local health officials and affected institutions is critical to avoiding contradictions and confusion that can undermine public trust and impede containment measures. To build public trust and cooperation, provide continuous, accurate, and science-based information on what is known and not known about the disease. Information should be technically correct and sufficiently complete to support policies and actions without being patronizing. Minimize duplication of, and ensure coordination between federal, state, local, and tribal authorities. **Avoid speculation**. During an outbreak, limit officially disseminated information to specific data and results; messages should omit speculation, over-interpretation of data, overly confident assessments of investigations and control measures, and comments related to other jurisdictions. Rumors, misinformation, misperceptions, and stigmatization of affected groups must be addressed promptly and definitively. **Provide safety guidelines**. It is essential to provide guidance to the public on actions to take to protect themselves and their family members and colleagues. Assess healthcare system cybersecurity and develop alternative plans for any cyberincident. **Institute travel limitations and screening**. Implement appropriate travel restrictions and airport screening to contain the international spread of an emerging infection. Airport screening may include passive passenger screening using questionnaires or sophisticated infrared equipment to screen all passengers for fever and indications of possible exposure, as well as health worker-conducted interviews. **Implement early and consistently support containment, testing, and aggressive contact tracing**. In the absence of a curative drug or preventive vaccine, well-known public health interventions can effectively contain an outbreak. The methods include active surveillance of suspected contacts, self-surveillance by contacts who voluntarily isolated themselves, and widespread testing, social distancing, and quarantine. **Stockpile necessary medications and equipment**. Enhance the national capability to produce and effectively use both medical countermeasures and nonpharmaceutical interventions, including those needed for both the acute and the chronic conditions. **Bolster national healthcare infrastructures**. A high priority is improving existing healthcare systems’ weaknesses that permit emerging infections to amplify and spread and that can compromise patient care. This includes having adequate materials and capacity for expected surges of infected patients, including hospitals and other healthcare facilities. **Protect healthcare workers**. The people at greatest risk for contracting the disease are health workers, including first responders. This requires working with professional societies to improve strategies (including PPE use) to protect healthcare workers. Special vigilance must be paid to women, who staff the lower ranks of health personnel in many countries. **Do just-in-time professional education**. Educate healthcare workers and public health staff on appropriate strategies to recognize the disease and to implement control measures. **Prepare the public**. Recognize that preparation for and control of pandemics are extremely disruptive and consume enormous resources at levels that might not be sustainable over time.

*WHO*, World Health Organization; *SARS*, severe acute respiratory syndrome; *PPE*, protective personal equipment.
